# HER2-directed therapy is associated with an increased rate of cardiologic emergency department visits in real-world breast cancer patients

**DOI:** 10.1016/j.breast.2026.104795

**Published:** 2026-05-02

**Authors:** Sandra Mayer, Anna Pfarrhofer, Sophie M. Neubauer, Sabina Pasalic, Georg Jeryczynski, Maximilian Marhold, Gerwin Heller, Jutta Bergler-Klein, Andreas Spannbauer, Thorsten Fuereder, Filippo Cacioppo, Anton Laggner, Matthias Preusser, Rupert Bartsch, Christoph Minichsdorfer

**Affiliations:** a Division of Oncology, Department of Internal Medicine I, Medical University of Vienna, Waehringer Guertel 18-201090, Vienna, Austria; b Division of Cardiology, Department of Internal Medicine II, Medical University of Vienna, Waehringer Guertel 18-201090, Vienna, Austria; c Department of Emergency Medicine, Medical University of Vienna, Waehringer Guertel 18-201090, Vienna, Austria

## Abstract

**Background:**

Subtype is a key prognostic factor for breast cancer. In HER2-positive disease, HER2-directed therapies have improved outcomes, though they can cause cardiac side effects, potentially leading to emergency department (ED) visits.

**Objectives:**

To assesses reasons for ED presentations in breast cancer patients, highlighting subtype-specific differences in reason for presentation, 3-month mortality (3 MM) and the possible association of cardiologic visits with HER2-directed therapy.

**Methods:**

In this retrospective study, visits by breast cancer patients at an Austrian tertiary care ED were analysed. Subtype frequency rates and subtype-specific 3 MM rates were calculated using Chi-Square tests (separately for early and advanced disease). A possible association between HER2-directed therapies and cardiologic ED visits was investigated using a Fisher's exact test and a multinomial logistic regression controlling for age.

**Results:**

There was a total of 463 ED visits among 322 patients between August 2016 and December 2019. Subtype distribution was as follows: 42% (n = 135) luminal B-like/HER2-negative, 23% (n = 74) triple negative, 16% (n = 50) luminal B-like/HER2-positive, 10% (n = 33) luminal A-like and 9% (n = 30) HER2-positive (non-luminal). In patients with advanced BC, subtype was significantly associated with 3 MM (p = 0.006), with the highest mortality rate observed in TNBC (54%). Active HER2-directed therapy (n = 70) was associated with increased cardiologic ED visits (OR = 4.536 [95%CI, 1.850- 11.125]).

**Conclusions:**

BC subtype influenced the frequency of ED visits and patient survival. HER2-directed therapy was associated with an increased risk for cardiologic ED visits among real-world cancer patients, emphasizing the need for tailored cardio-oncologic care strategies to optimize tolerability and reduce healthcare burdens.

## Highlights

•Frequency of and reasons for ED visits in breast cancer patients varied by subtype.
• HER2-directed therapy linked to higher rate of cardiologic emergency presentations.
• In advanced breast cancer, subtype influenced short-term mortality after ED visits.
• Mortality rates were highest in TNBC, HER2+ and Luminal B/HER2-negative patients.

## 1 Introduction

Breast cancer is a biologically heterogenous disease [1] and its molecular subtypes harbour major differences in biological background and treatment response and therefore act as important prognostic and predictive factors [[2], [3], [4], [5]]. Furthermore, subtype influences the pattern and timing of metastatic spread [[6], [7], [8], [9]] and informs about available treatment options [[10], [11], [12]].

For HER2-positive cancer, the emergence of HER2-directed therapies revolutionised treatment strategies and has led to a significantly improved prognosis [13,14]. As life expectancy increases, (long-term) tolerability becomes increasingly important. In this context, the known cardiotoxicity of HER2-directed therapies is a key concern [15]. While the cardiotoxic effects of these treatments have been well-documented in clinical studies, less data is available on their impact in real-world populations of patients with breast cancer, particularly regarding unplanned emergency department (ED) visits. Additionally, other novel treatment options may lead to clinically relevant toxicities as well and the side effects of conventional chemotherapy remain a concern. Finally, disease progression in patients with metastatic breast cancer may result in increased symptom burden and more frequent ED visits. Given the large number of annual cancer-related ED visits, among which breast cancer is one of the most common cancer diagnoses [16], a deeper understanding of breast cancer-related ED visits is crucial. Therefore, this retrospective analysis aimed at analysing ED presentations in a real-world collective of patients with breast cancer, focusing on possible association between HER2-directed therapies and cardiologic ED visits.

## 2 Methods

### 2.1 Study design

This study was designed as an explorative, retrospective evaluation of ED visits by patients with breast cancer treated at the ED of the Medical University of Vienna from 1st August 2016 to 31st December 2019.

This study was approved by the ethics committee of the Medical University of Vienna (EC No. 1446/2022).

### 2.2 Study collective

Eligible patients were aged ≥18 years, had an active diagnosis of early-stage or advanced breast cancer (or were undergoing adjuvant treatment after curative surgery). Patients receiving adjuvant endocrine therapy only or those with a second active primary malignancy were excluded. Cases with unknown data regarding the hormone receptor status or HER2 status were also excluded. Patients without a recorded histological type (lobular vs. non-lobular) were assigned to the non-lobular group. The selection process for the final study cohort is shown in Supplemental Fig. 1.

### 2.3 Data collection

Study data was retrospectively extracted from electronic medical records. We extracted patient demographics (age, sex, weight), cancer characteristics (histologic type, ER/PR-status, HER2 status, Ki67 according to the last available records in the medical files), clinical parameters (distant metastases, treatment setting, recent administration of systemic anti-cancer drugs at time of presentation) and data concerning the ED visit (admission diagnosis, discharge diagnosis, reason for visit [tumor-related, treatment-related, other cause], inpatient admission, length of in-hospital stay, and all-cause 3-month-mortality after initial ED presentation).

Patients were categorized into the subtypes luminal A-like, luminal B-like/HER2-negative, luminal B-like/HER2-positive, HER2-positive (non-luminal) and triple negative [17]. For subtype categorisation clinico-pathological surrogate subtypes as per St. Gallen 2013 were used, as data on the intrinsic subtype according to molecular signatures was not available. Cancers with <20% Ki67 expression were considered Ki67-low, all others as Ki67-high.

Histological type was categorized dichotomously as either lobular or non-lobular cancers. Where histological reports were unavailable, subtype and histological type were derived from chart entries and discharge letters.

Admission and discharge diagnoses were grouped into categories as outlined in Supplemental Tables 1 and 2 During our evaluation of admission diagnoses, we additionally assessed the frequency of “potentially preventable ED visits” (PPEDs), using the CMS diagnosis-based definition, which defines visits due to anemia, nausea, fever, dehydration, neutropenia, diarrhea, pain, pneumonia, sepsis, or emesis as potentially preventable [18].

### 2.4 Statistical analysis

IBM SPSS 29.0.0.0 was used for all statistical calculations.

For patients with multiple ED visits during the study period, individual ED visits were treated as stochastically independent observations; when analyses were patient-based, only the first visit per patient was included.

Categorical variables were summarized as absolute (n) and relative frequencies (%); continuous variables as medians, interquartile ranges, minimums and. Where p-values were calculated, a significance level of p < 0.05 was set.

The possible association between subtype and reasons for visit was evaluated using a Chi-Square test. To account for the confounders age, treatment setting and recent treatment administration, a multinomial logistic regression was performed, using these parameters as well as subtype as the independent variables and reason for visit as the dependent variable. For assessment of an association between cardiologic ED visits and HER2-directed treatment, discharge diagnoses were categorized dichotomously as “cardiologic” or “non-cardiologic”. Table 1 shows which discharge diagnoses were included into the “cardiologic” category. (Cardiologic diagnoses were not pre-defined but exploratory. Classification was performed manually based on the investigators' clinical judgment.) A Fisher's Exact test was performed, and the Odds Ratio (OR) calculated. To counteract a possible bias due to interdependence of multiple visits by the same patient, we repeated the calculations using only the first ED visit of each patient, as a sensitivity analysis. To account for age as a possible confounder, a multinomial logistic regression with discharge diagnosis (cardiologic vs. non-cardiologic) as the dependent variable and age and HER2-directed treatment (yes/no) as the independent variables was then added and the OR with its corresponding 95% CI calculated.

**Table 1:** Included discharge diagnoses categorized as “Cardiologic” (exploratory).

Group	Included diagnoses
Heart Failure and Left Ventricular Dysfunction	cardiac decompensation, cardiomyopathy, heart failure
Arrhythmias	tachyarrhythmic atrial fibrillation, atrial flutter, palpitations, atrioventricular nodal reentry tachycardia, tachycardia (not further specified)
Ischemic Events	myocardial infarction
Blood Pressure-Related Issues	hypertensive derailment
Other Cardiovascular Events	aortic dissection, cardiac tamponade

A possible association between anthracycline therapy and cardiologic ED visits was evaluated using a Fisher's exact test.

Three-month mortality rates were calculated separately for eBC and aBC patients and compared using a Chi-Square test.

Due to the exploratory and retrospective design of this study, no formal sample-size calculation or adjustment for multiple testing was performed, and results are therefore descriptive by nature and should be interpreted as hypothesis-generating.

## 3 Results

### 3.1 Study collective

After applying the inclusion and exclusion criteria, there was a total of 463 ED visits by 322 patients. 319/322 (99.1%) of patients were female and 3/322 (0.9%) male. Median age was 62 years (range 25-96 years) (Table 2).

**Table 2:** Baseline characteristics of included patients, stratified by tumor subtype.

		All	Luminal A-like	Luminal B-like/HER2-negative	Luminal B-like/HER2-positive	HER2-positive	Triple Negative
**number (%)**		322	33	135	50	30	74
		(100.0 %)	(10.2 %)	(41.9 %)	(15.5 %)	(9.3 %)	(23.0 %)
**sex**	female	319					
		(99.1 %)					
	male	3					
		(0.9 %)					
**median age**		62	67	65	57	56	58
**histologic type**	non-lobular	278	22	107	46	30	73
		(86.3%)	(66.7%)	(79.3%)	(92.0%)	(100.0%)	(98.6%)
	lobular	44	11	28	4	0	1
		(13.7%)	(33.3%)	(20.7%)	(8.0%)	(0.0%)	(1.4%)
**metastases**	no	100	13	33	18	11	25
		(31.1%)	(39.4%)	(24.4%)	(36.0%)	(36.7%)	(33.8%)
	yes	222	20	102	32	19	49
		(68.9%)	(60.6%)	(75.6%)	(64.0%)	(63.3%)	(66.2%)
**metastatic sites**^a^	bone	146	18	76	22	8	22
		(65.8%)	(90.0%)	(74.5%)	(68.8%)	(42.1%)	(44.9%)
	lung	78	6	29	11	8	24
		(35.1%)	(30.0%)	(28.4%)	(34.4%)	(42.1%)	(49.0%)
	liver	80	3	45	10	6	16
		(36.0%)	(15.0%)	(44.1%)	(31.3%)	(31.6%)	(32.7%)
	brain	48	1	11	7	10	19
		(21.6%)	(5.0%)	(10.8%)	(21.9%)	(52.6%)	(38.8%)
	peritoneal	19	4	8	1	1	5
		(8.6%)	(20.0%)	(7.8%)	(3.1%)	(5.3%)	(10.2%)
	other	53	4	29	6	3	11
		(23.9%)	(20.0%)	(28.4%)	(18.8%)	(15.8%)	(22.4%)
**treatment setting**	curative	93	11	30	18	10	24
		(28.9%)	(33.3%)	(22.2%)	(36.0%)	(33.3%)	(28.9%)
	palliative	229	22	105	32	20	50
		(71.1%)	(66.7%)	(77.8%)	(64.0%)	(66.7%)	(67.6%)
**reason for visit**	treatment-related	71	5	25	11	11	19
		(15.3%)	(12.5%)	(12.6%)	(14.1%)	(25.6%)	(18.3%)
	tumor-related	178	7	80	32	18	41
		(38.4%)	(17.5%)	(40.4%)	(41.0%)	(41.9%)	(39.4%)
	other cause	214	28	93	35	14	44
		(46.2%)	(70.0%)	(47.0%)	(44.9%)	(32.6%)	(42.3%)
**ongoing treatment**	no	159	13	71	22	15	38
		(34.3%)	(32.5%)	(35.9%)	(28.2%)	(34.9%)	(36.5%)
	yes	304	27	127	56	28	66
		(65.7%)	(67.5%)	(64.1%)	(71.8%)	(65.1%)	(63.5%)
**discharge**	outpatient	166	19	67	31	18	31
		(35.9%)	(47.5%)	(33.8%)	(39.7%)	(41.9%)	(29.8%)
	inpatient	297	21	131	47	25	73
		(64.1%)	(52.5%)	(66.2%)	(60.3%)	(58.1%)	(70.2%)

a Among advanced BC patients only.

Baseline characteristics including demographics, metastatic status, metastatic sites, treatment intent, ongoing therapy and discharge status are presented in Table 2.

### 3.2 Subtype distribution

Among 322 patients, luminal B-like/HER2-negative was the most common subtype (135 patients, 41.9%), followed by triple negative (74 patients, 23%), luminal B-like/HER2-positive (50 patients, 15.5%), luminal A-like (33 patients, 10.2%) and HER2-positive (non-luminal) (30 patients, 9.3%). Concerning histological subtype, there were 44/322 patients with lobular breast cancer (13.7%).

### 3.3 Admission & discharge diagnoses

The most common admission diagnoses among all 463 visits were pain (N = 106, 22.9%), respiratory symptoms (N = 94, 20.3%), fever, malaise (N = 56, 12.1% each), neurologic symptoms (N = 49, 10.6%) and GI complaints (N = 43, 9.3%). These six symptom groups accounted for >80% of all admission diagnoses. Table 3 presents frequency rates of the most common admission diagnoses in dependence of treatment intent. A complete list of frequency rates of all admission diagnosis groups according to subtype can be found in Supplemental Table 4.

**Table 3:** Comparison of the frequency rates of the most common admission diagnoses between patients with early (eBC) and advanced (aBC) breast cancer.

	eBC	aBC
Fever	17.3 %	10.0 %
Gastrointestinal symptoms	12.0 %	8.2 %
Pain	25.6 %	21.8 %
Respiratory symptoms	12.0 %	23.6 %
Malaise	6.8 %	14.2 %
Neurologic symptoms	9.0 %	11.2 %

When applying the Centers for Medicare & Medicaid Services (CMS) definition of potentially preventable ED visits [18], we found that a total of 217/463 visits (46,9%) could be defined as potentially preventable (for an exact listing of frequencies for each diagnosis see Supplemental Table 5).

The most common overall discharge diagnoses were “Cancer” (93/463 visits, 20.1%), “Infection” (87/463 visits, 18.8%), “Edema/Effusion” (50/463 visits, 10.8%), “Pain” (36/463 visits, 7.8%) and “GI” (26/463 visits, 5.6%). Table 4 presents the top 5 discharge diagnoses for each subtype; a complete list of frequencies of all discharge diagnoses per subtype can be found in Supplemental Table 6.

**Table 4:** Top 5 discharge diagnoses per subtype and their respective relative frequencies.

	Luminal A-like	Luminal B-like/HER2-negative	Luminal B-like/HER2-positive	HER2-positive (non-luminal)	Triple Negative
1.	Infection (27.5%)	Cancer (19.2%)	Cancer (26.9%)	Infection (25.6%)	Cancer (23.1%)
2.	Cancer (10%)	Infection (17.2%)	Infection (19.2%)	Cancer (14%)	Infection (15.4%)
3.	Pain (10%)	Edema/Effusion (13.6%)	Edema/Effusion (11.5%)	Cardiologic (11.6%)	GI (8.7%)
4.	Neurologic (7.5%)	Pain (8.6%)	Cardiologic (9%)	Pain (11.6%)	Edema/Effusion (7.7%)
5.	Respiratory (7.5%)	GI (6.1%)	Pain (5.1%)	Edema/Effusion (9.3%)	Malaise (5.8%)

### 3.4 Cardiologic ED visits and HER2-directed therapy

**Fig. 1:**
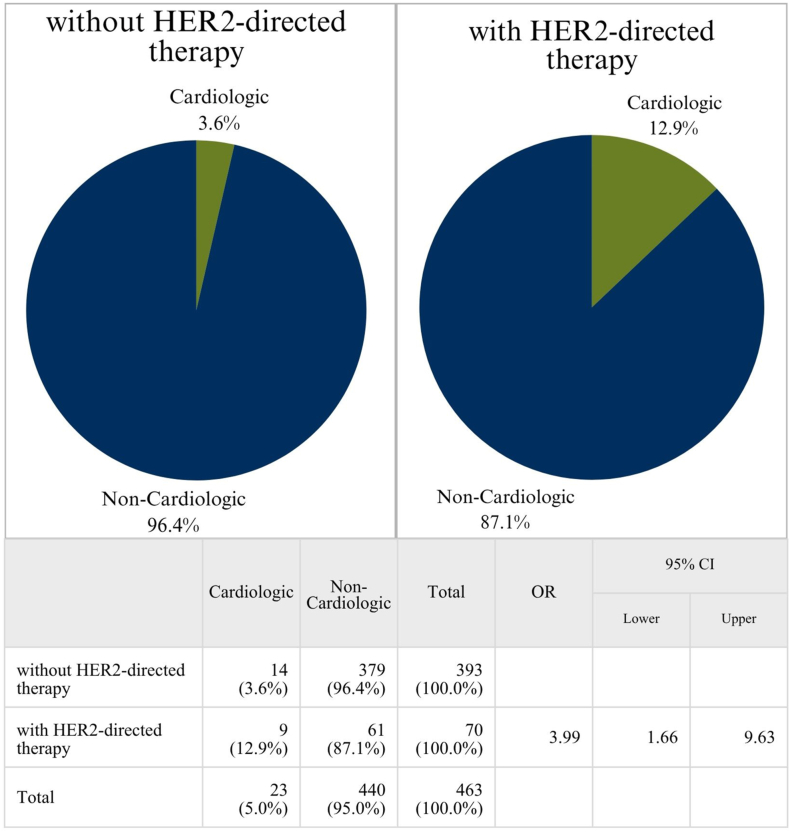
**Association between HER2-directed therapy and cardiologic ED visits.** Proportion of cardiologic vs. non-cardiologic ED visits in patients with and without HER2-directed therapy, as illustrated with two pie charts. Cardiologic visits occurred more frequently in the group receiving HER2-directed therapy (12.9% vs. 3.6%). The accompanying table shows the corresponding Odds Ratio after accounting for age (OR = 3.994 [95% CI, 1.657- 9.629]) as well as absolute and relative frequencies. This association between HER2-directed therapy and increased cardiologic ED visits points to a possible need for more intensive cardio-oncologic monitoring in this group, though prospective validation is needed.

Among 70 patients undergoing HER2-directed therapy, 44 had received trastuzumab-pertuzumab, 16 had received trastuzumab-mono therapy, 7 had received trastuzumab-emtansin and 3 had received lapatinib. Patients undergoing HER2-directed therapy were significantly more likely to present with cardiologic complaints than those who had not received such treatment (OR = 3.994 [95% CI, 1.657-9.629], p = 0.003). Fig. 1 illustrates this association using two pie charts and presents exact frequencies alongside the corresponding OR. After accounting for age in a multinomial logistic regression, the correlation remained statistically significant with p = 0.002 and OR = 4.536 [95% CI, 1.850- 11.125]). When restricting the analysis to only one ED visit per patient, the correlation between HER2-directed therapy and cardiologic visits remained statistically significant (OR = 3.876 [95% CI 1.339 -11.236], p = 0.018). 55.6% of cardiologic visits in HER2-treated patients resulted in inpatient admission. The cardiac events reported among HER2-treated patients included congestive heart failure, arrhythmias (atrial fibrillation, unspecified tachycardia) and hypertensive derailment.

40 patients had received anthracycline therapy. Though cardiologic ED visits were numerically more frequent among anthracycline-treated patients compared with those without anthracycline exposure (10.0% vs. 4.5%), this difference did not reach statistical significance (Fisher's exact test p = 0.127). Only 8 patients had received both HER2-directed therapy and anthracyclines and a Fisher's exact test revealed no statistically significant association with cardiologic ED visits for these patients (p = 0.337)

### 3.5 Reason for visit

Overall, 71/463 ED visits (15.3%) were treatment-related, 178/463 (38.4%) tumour-related, and 214/463 visits (46.2%) due to causes not directly related to the tumour or its treatment. After adjusting for confounders (age, treatment setting and recent treatment administration), a multinomial logistic regression found no significant association between subtype and reason for visit with χ 2 (8) = 13.317 and p = 0.10. There were however significantly fewer tumour-related visits in luminal A-like patients compared to patients with triple negative disease (OR = 0.31, p = 0.021).

### 3.6 Three-month mortality (3 MM)

In patients with early breast cancer (93 patients), two deaths within 3 months of the ED visit were recorded, both of which were unrelated to the oncologic disease. Thus, no significant association was found between subtype and 3 MM in this group (p = 0.43).

**Fig. 2:**
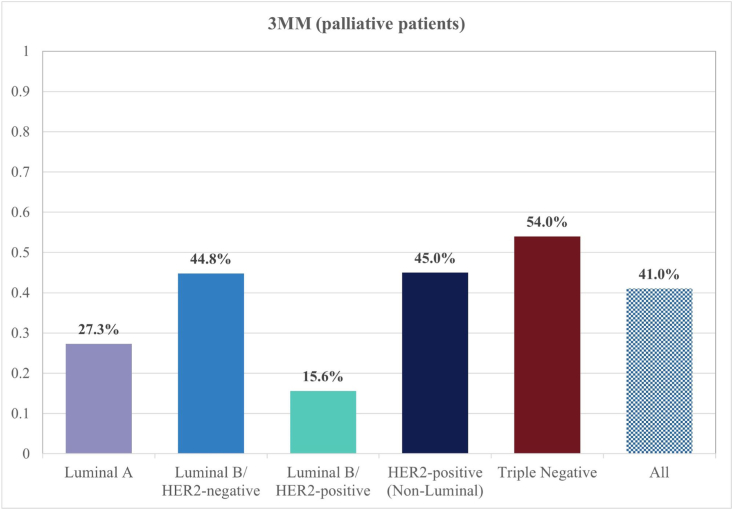
**Subtype-specific three-month mortality rates in palliative patients.** Bar chart presenting three-month mortality rates among 229 palliative patients. Each bar represents one subtype, while the bar to the far-right presents the mortality rate among all 229 patients. Mortality was highest in patients with triple-negative cancer (54%), followed by HER2-positive (45%) and luminal B-like/HER2-negative (44.8%) disease. In contrast, patients with luminal A-like (27.3%) and especially luminal B-like/HER2-positive subtype (15.6%) had significantly lower mortality rates. The overall three-month mortality across all subtypes was 41.0%. These findings underscore significant differences in short-term survival by molecular subtype, with implications for monitoring and emergency care planning.

In the advanced subgroup (229 patients) however, subtype was significantly associated with 3 MM (p = 0.006). As illustrated in Fig. 2, in the aBC patient population, the highest 3 MM rate was found in patients with triple negative disease, as 27/50 (54%) of these patients died within three months of their ED visit. Conversely, in the luminal B-like/HER2-positive subtype, 5/32 (15.6%) of patients died, making this subtype the one with the numerically best short-term outcome. Mortality rates for the remaining subtypes were as follows: HER2-positive (non-luminal) (9/20, 45%), luminal B-like/HER2-negative (47/105, 44.8%) and luminal A-like (6/22, 27.3%). The odds of dying within three months were 6.3 times 12 higher if the patient had TNBC compared with luminal B-like/HER2 positive disease [95% CI, 2.101-19.130].

Results of a subgroup-analysis on 3 MM-rates among patients with lobular cancer can be found in Supplemental Tables 7 and 8

## 4 Discussion

This retrospective study offers valuable insights into the influence of breast cancer subtype on ED utilization and subsequent short-term survival in a real-world population of patients with breast cancer. Notably, we identified a significantly increased risk of cardiologic ED visits in patients receiving HER2-directed therapies. Given the retrospective and exploratory nature of our study, our findings should however be interpreted as hypothesis-generating only.

### 4.1 Subtype distribution and 3 MM

The subtype distribution we found in our ED cohort differed substantially from the general population of breast cancer patients [19], as luminal B-like/HER2-negative was the most common subtype, followed by triple negative disease. Both subtypes were not only overrepresented but also exhibited highly unfavourable survival outcomes (with highest 3 MM rates of 54% in advanced TNBC patients). While the aggressive behaviour of TNBC (and its limited treatment options) are widely known [20], our findings for the luminal B-like/HER2-negative subtype were more noteworthy. Though comparing this subtype's frequency to the general breast cancer population is complicated by the use of varying classification systems for subtyping in previous epidemiologic studies (resulting in widely differing frequency rates being reported) [3,[21], [22], [23], [24]], its predominance in our analysis, coupled with survival rates only marginally better than those in non-luminal cancers, indicates a need for further investigation. As this subtype is often grouped together with luminal A-like cancers, its relatively more aggressive behaviour [[25], [26], [27]] may previously have been underestimated. Ultimately, both TNBC and luminal B-like/HER2-negative cancers may be in need of closer monitoring and timely tailored interventions to prevent ED visits and allow for more effective emergency care – pending further research, ideally in the prospective setting. The outcome for luminal B-like/HER2-positive patients on the other hand was comparatively favourable in our analysis, with 3 MM rates (15.4%) even lower than those in the generally less aggressive luminal A-like subtype (which is known for its favourable prognosis [28,29] and less toxic therapeutic options [11,12,30]) – likely due to the availability of highly effective targeted treatment combinations for this subtype, especially in the metastatic setting. When reflecting on the subtype distribution and mortality patterns observed in our study, it is important to acknowledge that patients presenting to the ED reflect a selectively sicker population than the general BC population and it is probable that this selection bias contributed to both the overrepresentation of certain subtypes and the short mortality rates we observed.

Our findings concerning admission diagnoses show that a vast majority of ED visits is caused by a relatively small set of symptoms (respiratory complaints, pain, fever, malaise, GI and neurologic symptoms). This may present an opportunity for targeted prevention strategies in routine oncologic care and the implementation of interventions before symptoms become so acute that they warrant an emergency visit – especially since we found that almost half of all visits could be defined as “potentially preventable” using the CMS definition. Due to the observed differences between eBC and aBC patients, a tailored approach could be beneficial.

### 4.2 HER2-directed therapy and cardiologic ED visits

Since the introduction of HER2-directed therapies such as trastuzumab, the historically unfavourable prognosis [2,5] of HER2-positive breast cancer has improved significantly [13,14]. Cardiologic side effects (spanning from asymptomatic LVEF decline to CHF) were reported in clinical trials of HER2-directed drugs [13,31]. In our study, we were able to highlight the possible real-world implications of these risks: We observed a four-fold increase in the odds of cardiologic ED visits in patients receiving HER2-directed therapy. The cardiologic discharge diagnoses found in patients who had received HER2-directed treatment in our study included congestive heart failure, cardiomyopathy, arrhythmias (atrial fibrillation, unspecified tachycardia) and hypertensive derailment. While the association between trastuzumab and CHF has been extensively characterized [32], the association between the other diagnoses included and HER2-targeted therapies is less clear. The 2022 ESC guidelines on cardio-oncology does however list both arrhythmias and hypertension as moderate risk factors for the occurrence of cancer therapy-related cardiovascular toxicity (CTR-CVT) [33] and the FDA package insert includes hypertension and arrhythmias as possible adverse events of HER2-directed therapy [34]. Due to the study design, a causal relation between these cardiologic ED visits and HER2-targeted therapy cannot be proven. Attribution bias should also be considered, as clinicians may be more likely to attribute symptoms to cardiotoxicity in the HER2-treated group. Still, our findings hint at the importance of careful patient selection, monitoring and, where necessary, the consideration of cardioprotective strategies for patients undergoing HER2-directed therapy. Closely adhering to the 2022 ESC guidelines on cardio-oncology [33] could aid in preventing some cardiologic events in patients undergoing therapy. It must be noted that the patients in this study were treated before publication of these guidelines. Moreover, it should be kept in mind that the treatment landscape for breast cancer has evolved since the study period of this project and novel HER2-directed therapies are now increasingly being used in patients with HER2-low triple negative and HR-positive/HER2-negative cancers as well [35,36]. As a result, the association of HER2-directed therapies and cardiologic ED visits we found in our study could become increasingly relevant for these other subtypes as well.

### 4.3 Limitations

The study's retrospective design may affect the accuracy and generalizability of the findings due to the possibility of missing data in the medical files and transcription errors occurring with manual data extraction. The retrospective nature also does not allow the definitive determination of a causal connection between cancer subtypes, ED visits, and survival outcomes. For the calculations concerning the link between cardiologic ED visits and HER2-directed therapies, “cardiologic” admission diagnoses were defined post hoc, were exploratory in nature, and not independently validated. Therefore, some of the included diagnoses may not reliably indicate cardiotoxicity. Additionally, there was no baseline cardiac assessment and our data set did not include factors such as comorbidities, smoking and further cardiovascular risk factors. These factors were outside of the scope of this study but could act as possible confounders. Small subgroup sizes limit statistical power and treating multiple visits by the same patient as independent could affect statistical accuracy. Though we repeated the analyses using only the first ED visit per patient as a sensitivity analysis, we acknowledge that more sophisticated methods to account for interdependence of repeated visits exist. The use of Ki67 as a marker for luminal differentiation is debated. While we were able to unveil possible associations and trends, further prospective studies with larger cohorts are needed to validate and expand upon our findings. Lastly, this analysis was conducted at a single tertiary care facility – since practice patterns may vary across institutions, this could also limit the generalizability of our data. Moreover, there was no local institutional or national data available on general subtype distribution, so direct comparison with a representative population from the same region was not possible and we had to rely on international data to put our findings into context.

## 5 Conclusions

The results of this study reveal key factors – such as HER2-directed therapies, triple negative and luminal B-like/HER2-negative subtype – that apparently influence ED presentations among breast cancer patients. For affected patients closer monitoring and the implementation of preventative measures might be warranted. Given that many of these ED visits may be preventable with adequate care strategies, this could be an opportunity for reducing ED utilization and associated costs, while also improving patients’ quality of life.

## CRediT authorship contribution statement

**Sandra Mayer:** Writing – original draft, Visualization, Project administration, Investigation, Formal analysis, Data curation. **Anna Pfarrhofer:** Data curation. **Sophie M. Neubauer:** Data curation. **Sabina Pasalic:** Data curation. **Georg Jeryczynski:** Writing – review & editing, Data curation. **Maximilian Marhold:** Writing – review & editing. **Gerwin Heller:** Writing – review & editing. **Jutta Bergler-Klein:** Writing – review & editing. **Andreas Spannbauer:** Writing – review & editing. **Thorsten Fuereder:** Writing – review & editing, Methodology. **Filippo Cacioppo:** Writing – review & editing, Resources, Methodology. **Anton Laggner:** Writing – review & editing, Resources, Data curation. **Matthias Preusser:** Writing – review & editing, Supervision, Data curation. **Rupert Bartsch:** Writing – review & editing, Supervision, Data curation, Conceptualization. **Christoph Minichsdorfer:** Writing – original draft, Supervision, Resources, Project administration, Methodology, Investigation, Data curation, Conceptualization.

## Declaration of competing interest

Thorsten Fuereder has received honoraria for lectures, consultation, advisory board participation or support for travel/accommodation/expenses from the following for-profit companies: Merck KGaA, Amgen, Boehringer Ingelheim, Janssen, Takeda, Invios, Lilly, BeiGene, PharmaMar, MSD, Daiichi Sankyo/Astra Zeneca, Bristol-Mayers Squibb/Celgene, Roche, Pfizer, Pierre Fabre, Kura Oncology, MSD, Bristol-Myers, Squibb/Celgene and Merck KGaA. Matthias Preusser has received honoraria for lectures, consultation or advisory board participation from the following for-profit companies: Bayer, Bristol-Myers Squibb, Novartis, Gerson Lehrman Group (GLG), CMC Contrast, GlaxoSmithKline, Mundipharma, Roche, BMJ Journals, MedMedia, Astra Zeneca, AbbVie, Lilly, Medahead, Daiichi Sankyo, Sanofi, Merck Sharp & Dome, Tocagen, Adastra, Gan & Lee Pharmaceuticals, Janssen, Servier, Miltenyi, Böhringer-Ingelheim, Telix, Medscape, OncLive, Nerviano Medical Sciences, ITM Oncologics GmbH.

Rupert Bartsch has received honoraria for lectures, consultation, advisory board participation support for travel/accommodation/expenses from the following for-profit companies: AstraZeneca, Daiichi Sankyo, Eisai, Lilly, MSD Oncology, Novartis, Pfizer, Pierre Fabre, Puma Biotechnology, Roche, Seagen.

Christoph Minichsdorfer has received honoraria for lectures, consultation, advisory board participation or support for travel/accommodation/expenses from the following for-profit companies: MSD, Merck, Roche, Sandoz.

All remaining authors have declared no conflicts of interest.
