# Supplementary material for: HER2-directed therapy is associated with an increased rate of cardiologic emergency department visits in real-world breast cancer patients
Source: Breast. 2026 May 2;88:104795. doi: 10.1016/j.breast.2026.104795 (PMC13158383; doi:10.1016/j.breast.2026.104795)
Supplement: Multimedia component 1 [file mmc1.docx]

# Supplementary Material

Supplemental Figure 1: Study cohort selection flowchart

screened ED visits

[N = 7.137]

no active malignancy/
insufficient data/
inclusion criteria not met

[N = 6.661]

subtype unknown

[N = 13 visits]

by

[n = 12 patients]

final sample size

[N = 463 visits]

by

[n = 322 patients]

Supplemental Table 1: Admission diagnoses included in each superordinate diagnosis group (post-hoc)

| **Category** | **Included Admission Diagnoses** |
| --- | --- |
| **Allergic Reaction** | Contrast agent allergy |
| **Bleeding** | Bleeding (not further specified), Hematemesis, Hematochezia, Hemoptysis, Hemorrhoids, Petechiae, Tumor bleeding, Vaginal bleeding |
| **Blood Clots** | Pulmonary embolism |
| **Cardiologic** | Cardiac arrest, Hypertension, Cardiogenic shock, Palpitations, Tachycardia |
| **Control Visit** | Control visit |
| **Dermatologic** | Erythema, Skin lesions, Rash, Ulceration |
| **Edema/Effusion** | Arm swelling, Ascites, Leg edema, Pleural effusion |
| **Exsiccosis** | Exsiccosis |
| **Fever** | Fever, Chills |
| **Gastrointestinal (GI)** | Diarrhea, Jaundice, Nausea & Vomiting, Constipation |
| **Injury** | Fall |
| **Medical Device Complication** | Medical device complication (not specified), Port-a-cath dislocation |
| **Laboratory Abnormalities** | Hyperglycemia |
| **Malaise** | Deterioration in general condition, General weakness |
| **Neurologic** | Aphasia, Cerebral space-occupying lesion, Confusion, Dizziness, Muscle cramping, Neurologic abnormalities (not specified), Paresthesia, Polyneuropathy, Seizure, Somnolence, Syncope, Vertigo, Speech disturbance |
| **Pain** | Abdominal pain, Chest pain, Full-body pain, Headache, Musculoskeletal pain, Neck pain, Lower back pain |
| **Psychiatric** | Anxiety, Panic attack |
| **Respiratory** | Dyspnea, Cough, Hyperventilation |
| **Surgical** | Wound check, Wound complication |
| **Urologic** | Urinary retention |

Supplemental Table 2: Discharge diagnoses included in each superordinate diagnosis group

| **Category** | **Included Discharge Diagnoses** |
| --- | --- |
| **Allergic Reaction** | Anaphylaxis, G-CSF overshoot, Contrast agent allergy, Intolerance reaction |
| **Bleeding** | Gastrointestinal hemorrhage, Hematoma, Intracranial hemorrhage, Tumor hemorrhage, Wound bleeding |
| **Blood Clots** | Intestinal ischemia, Cerebral venous sinus thrombosis, PAC thrombosis, Deep vein thrombosis (DVT), Pulmonary embolism |
| **Cancer** | Metastases, Tumor progression, Breast cancer (initial diagnosis, recurrence), Malignant pleural effusion, Tumor pain, Tumor-related nausea |
| **Cardiologic** | Aortic dissection, AVNRT, Hypertensive derailment, Cardiac decompensation, Cardiomyopathy, Palpitations, Myocardial infarction, Cardiac tamponade, Tachycardia, Atrial fibrillation, Atrial flutter |
| **Dehydration** | Dehydration, Hypovolemia |
| **Dermatologic** | Erysipelas, Erythema, Contact eczema, Stasis dermatitis, Ulceration, Urticaria |
| **Edema/Effusion** | Ascites, Leg edema, Cerebral edema, Pulmonary edema, Pleural effusion |
| **Electrolyte Imbalance** | Hyponatremia, Hypercalcemia |
| **Endocrinological** | Blood sugar dysregulation, SIADH, Hypoglycemia |
| **Gastrointestinal (GI)** | Chemotherapy-induced nausea and emesis, Choledocholithiasis, Bowel perforation, Diarrhea (including chemotherapy-related), Dyspepsia, Emesis, Hemorrhoids, Intestinal adhesions, Constipation |
| **Hematologic** | Anemia, Thrombopenia, Hemolysis, Leukopenia, (Febrile) Neutropenia, Pancytopenia |
| **Infection** | Agranulocytosis, Vaginitis, Bacteremia, Bronchitis, Cholangitis, Cholecystitis, Clostridium infection, Colitis, Enteritis, Fever, Infection (various types, including post-chemotherapy), Influenza, Mastitis, Mucositis, Pneumonia, Sepsis, Soft tissue infection, Wound infection |
| **Intoxication** | Digitoxin overdose, Intoxication (unspecified) |
| **Malaise** | General weakness, Deterioration in general condition, Fatigue |
| **Medical Device Complication** | Medical Device Complication |
| **Nephrological** | Acute renal failure, Chronic kidney disease |
| **Neurologic** | Benign paroxysmal positional vertigo, Collapse, Polyneuropathy, Seizure, Stroke, Carpal tunnel syndrome, Dizziness, Syncope, Transient ischemic attack (TIA) |
| **Orthopedic** | Fractures, Empyema, Lumbago, Osteomyelitis |
| **Pain** | Abdominal pain, Full-body pain, Bone pain, Headache, Musculoskeletal pain, Chest pain |
| **Psychiatric** | Adjustment disorder, Hyperventilation syndrome, Panic attack, Psychogenic chest pain |
| **Respiratory** | COPD, Dyspnea, Pneumothorax, Chronic cough, Respiratory failure |
| **Stomatology** | Incomplete jaw lock |
| **Urologic** | Obstructive uropathy |

Supplemental Table 3: Reasons for visit included in the categories "tumor-related", "treatment-related" and "other cause"

| **Category** | **Included reasons for visit** |
| --- | --- |
| Tumor-related ^a^ | initial tumor diagnosis, tumor progress, tumor bleeding, tumor pain, tumor fever, tumor recurrence, malignant effusion (i.e. ascites, pericardial or pleural effusion), tumor lysis syndrome, hypercalcemia, hyponatremia, anemia, (febrile) neutropenia, thrombopenia, hyperviscosity syndrome, hyperleukocytosis and leukostasis, superior vena cava syndrome, mechanical ileus, malignant spinal cord compression, cerebral edema, increased intracranial pressure, acute obstructive hydrocephalus, seizures and any neurological deficits due to brain metastases or leptomeningeal carcinomatosis, cerebral venous sinus thrombosis, malignant airway obstruction, hemoptysis, pathological fractures |
| Treatment-related ^b^ | anaphylaxia and other allergic reactions, extravasation reactions, injection site reactions, exanthema, nausea, emesis, diarrhea, bowel perforation, acute liver failure, hyperglycemia, anemia, leukopenia, (febrile) neutropenia, thrombopenia, pancytopenia, fever, hemorrhage, cardiomyopathy, congestive heart failure, coronary vasospasms, myocarditis, pericarditis, arterial and venous thromboembolism, arrhythmias, thrombotic microangiopathy, hypertensive crisis, acute kidney injury, malaise and fatigue; immune-related events like colitis, mucositis, oesophagitis, pneumonitis, hepatitis, hypophysitis; cytokine release syndrome, adrenal insufficiency, polyneuropathy, skin reactions (i.e. dermatitis, edema, ulceration, local infection), arthralgia, myalgia and musculoskeletal pain in general, headache, fluid retention, ototoxicity |
| Other cause | any other reasons for emergency visits, which were neither causally connected to the cancer, nor to its treatment |

^a^ ED visits were only considered tumor-related if a causal association between the described symptoms and the tumor itself or its metastases could plausibly be made. (i.e. in case of malignant bone marrow infiltration for neutropenias and thrombopenias).

^b^ ED visits were only considered treatment-related if a causal association between the recent administration of anti-tumor-treatment and the respective symptom could plausibly be made (i.e. emesis shortly after chemotherapy administration).

Supplemental Table 4: Absolute and relative frequencies of all admission diagnosis groups by breast cancer subtypes

|  | All | Luminal A | Luminal B/HER2-negative | Luminal B/HER2-positive | HER2-positive | Triple Negative |
| --- | --- | --- | --- | --- | --- | --- |
| Pain | 106 | 11 | 40 | 27 | 11 | 17 |
|  | (22,9%) | (27,5%) | (20,2%) | (34,6%) | (25,6%) | (16,3%) |
| Respiratory | 94 | 6 | 49 | 15 | 8 | 16 |
|  | (20,3%) | (15,0%) | (24,7%) | (19,2%) | (18,6%) | (15,4%) |
| Fever | 56 | 5 | 21 | 7 | 9 | 14 |
|  | (12,1%) | (12,5%) | (10,6%) | (9,0%) | (20,9%) | (13,5%) |
| Malaise | 56 | 0 | 29 | 7 | 3 | 11 |
|  | (12,1%) | (0,0%) | (14,6%) | (9,0%) | (7,0%) | (10,6%) |
| Neurologic | 49 | 5 | 15 | 8 | 6 | 15 |
|  | (10,6%) | (12,5%) | (7,6%) | (10,3%) | (14,0%) | (14,4%) |
| GI | 43 | 3 | 18 | 6 | 2 | 14 |
|  | (9,3%) | (7,5%) | (9,1%) | (7,7%) | (4,7%) | (13,5%) |
| Bleeding | 15 | 1 | 7 | 0 | 2 | 5 |
|  | (3,2%) | (2,5%) | (3,5%) | (0,0%) | (4,7%) | (4,8%) |
| Cardiologic | 10 | 1 | 5 | 1 | 1 | 2 |
|  | (2,2%) | (2,5%) | (2,5%) | (1,3%) | (2,3%) | (1,9%) |
| Edema/Effusion | 9 | 0 | 5 | 0 | 1 | 3 |
|  | (1,9%) | (0,0%) | (2,5%) | (0,0%) | (2,3%) | (2,9%) |
| Dermatologic | 6 | 0 | 4 | 1 | 0 | 1 |
|  | (1,3%) | (0,0%) | (2,0%) | (1,3%) | (0,0%) | (1,0%) |
| Surgical | 4 | 0 | 0 | 2 | 0 | 2 |
|  | (0,9%) | (0,0%) | (0,0%) | (2,6%) | (0,0%) | (1,9%) |
| Urologic | 3 | 0 | 1 | 0 | 0 | 2 |
|  | (0,6%) | (0,0%) | (0,5%) | (0,0%) | (0,0%) | (1,9%) |
| Blood clots | 2 | 0 | 1 | 1 | 0 | 0 |
|  | (0,4%) | (0,0%) | (0,5%) | (1,3%) | (0,0%) | (0,0%) |
| Controll visit | 2 | 0 | 0 | 0 | 0 | 0 |
|  | (0,4%) | (0,0%) | (0,0%) | (0,0%) | (0,0%) | (0,0%) |
| Psychiatric | 2 | 0 | 0 | 1 | 0 | 1 |
|  | (0,4%) | (0,0%) | (0,0%) | (1,3%) | (0,0%) | (1,0%) |
| Allergic reaction | 1 | 0 | 0 | 1 | 0 | 0 |
|  | (0,2%) | (0,0%) | (0,0%) | (1,3%) | (0,0%) | (0,0%) |
| Exsiccosis | 1 | 0 | 0 | 1 | 0 | 0 |
|  | (0,2%) | (0,0%) | (0,0%) | (1,3%) | (0,0%) | (0,0%) |
| Injury | 1 | 0 | 1 | 0 | 0 | 0 |
|  | (0,2%) | (0,0%) | (0,5%) | (0,0%) | (0,0%) | (0,0%) |
| Laboratory abnormalities | 1 | 0 | 1 | 0 | 0 | 0 |
|  | (0,2%) | (0,0%) | (0,5%) | (0,0%) | (0,0%) | (0,0%) |
| Medical device complication | 2 | 0 | 1 | 0 | 0 | 1 |
|  | (0,4%) | (0,0%) | (0,5%) | (0,0%) | (0,0%) | (1,0%) |
| Total | 463 | 40 | 198 | 78 | 43 | 104 |
|  | (100,0%) | (100,0%) | (100,0%) | (100,0%) | (100,0%) | (100,0%) |

Supplemental Table 5: Absolute and relative frequencies of
potentially preventable ED visit (as per the CMS classification)

| Diagnosis | Abs. Frequency | Relative frequency |
| --- | --- | --- |
| Anemia | 4 | 0,9% |
| Nausea/Emesis | 25 | 5,4% |
| Fever | 42 | 9,1% |
| Dehydration | 7 | 1,5% |
| Neutropenia | 10 | 2,2% |
| Diarrhea | 10 | 2,2% |
| Pain | 106 | 22,9% |
| Pneumonia | 9 | 1,9% |
| Sepsis | 3 | 0,6% |
| Total | 217 | 46,9% |

Note that 14 visits with the initial admission diagnosis “Fever”
were re-categorized to “Neutropenia” (9), “Sepsis” (3) and
“Pneumonia” (2) for this analysis upon further examination.

Supplemental Table 6: Absolute and relative frequencies of all discharge diagnosis groups by breast cancer subtypes

| **Discharge diagnosis** | **All** | **Luminal A** | **Luminal B/HER2-negative** | **Luminal B/HER2-positive** | **HER2-positive** | **Triple Negative** |
| --- | --- | --- | --- | --- | --- | --- |
| Cancer | 93 | 4 | 38 | 21 | 6 | 24 |
|  | (20,1%) | (10,0%) | (19,2%) | (26,9%) | (14,0%) | (23,1%) |
| Infection | 87 | 11 | 34 | 15 | 11 | 16 |
|  | (18,8%) | (27,5%) | (17,2%) | (19,2%) | (25,6%) | (15,4%) |
| Edema/Effusion | 50 | 2 | 27 | 9 | 4 | 8 |
|  | (10,8%) | (5,0%) | (13,6%) | (11,5%) | (9,3%) | (7,7%) |
| Pain | 36 | 4 | 17 | 4 | 5 | 6 |
|  | (7,8%) | (10,0%) | (8,6%) | (5,1%) | (11,6%) | (5,8%) |
| GI | 26 | 2 | 12 | 3 | 0 | 9 |
|  | (5,6%) | (5,0%) | (6,1%) | (3,8%) | (0,0%) | (8,7%) |
| Cardiologic | 23 | 1 | 9 | 7 | 5 | 1 |
|  | (5,0%) | (2,5%) | (4,5%) | (9,0%) | (11,6%) | (1,0%) |
| Blood clots | 18 | 0 | 11 | 2 | 2 | 3 |
|  | (3,9%) | (0,0%) | (5,6%) | (2,6%) | (4,7%) | (2,9%) |
| Malaise | 18 | 1 | 9 | 1 | 1 | 6 |
|  | (3,9%) | (2,5%) | (4,5%) | (1,3%) | (2,3%) | (5,8%) |
| Neurologic | 17 | 3 | 6 | 2 | 4 | 2 |
|  | (3,7%) | (7,5%) | (3,0%) | (2,6%) | (9,3%) | (1,9%) |
| Hematologic | 11 | 1 | 4 | 1 | 0 | 5 |
|  | (2,4%) | (2,5%) | (2,0%) | (1,3%) | (0,0%) | (4,8%) |
| Bleeding | 10 | 0 | 5 | 1 | 2 | 2 |
|  | (2,2%) | (0,0%) | (2,5%) | (1,3%) | (4,7%) | (1,9%) |
| Dermatologic | 10 | 0 | 4 | 1 | 0 | 5 |
|  | (2,2%) | (0,0%) | (2,0%) | (1,3%) | (0,0%) | (4,8%) |
| Orthopedic | 9 | 2 | 4 | 1 | 0 | 2 |
|  | (1,9%) | (5,0%) | (2,0%) | (1,3%) | (0,0%) | (1,9%) |
| Other | 9 | 1 | 3 | 2 | 0 | 3 |
|  | (1,9%) | (2,5%) | (1,5%) | (2,6%) | (0,0%) | (2,9%) |
| Nephrological | 7 | 1 | 3 | 1 | 0 | 2 |
|  | (1,5%) | (2,5%) | (1,5%) | (1,3%) | (0,0%) | (1,9%) |
| Dehydration | 6 | 1 | 2 | 0 | 1 | 2 |
|  | (1,3%) | (2,5%) | (1,0%) | (0,0%) | (2,3%) | (1,9%) |
| Psychiatric | 6 | 0 | 0 | 2 | 2 | 2 |
|  | (1,3%) | (0,0%) | (0,0%) | (2,6%) | (4,7%) | (1,9%) |
| Respiratory | 5 | 3 | 1 | 0 | 0 | 1 |
|  | (1,1%) | (7,5%) | (0,5%) | (0,0%) | (0,0%) | (1,0%) |
| Electrolyte imbalance | 4 | 1 | 1 | 1 | 0 | 1 |
|  | (0,9%) | (2,5%) | (0,5%) | (1,3%) | (0,0%) | (1,0%) |
| Endocrinological | 4 | 0 | 4 | 0 | 0 | 0 |
|  | (0,9%) | (0,0%) | (2,0%) | (0,0%) | (0,0%) | (0,0%) |
| Allergic reaction | 3 | 0 | 1 | 1 | 0 | 1 |
|  | (0,6%) | (0,0%) | (0,5%) | (1,3%) | (0,0%) | (1,0%) |
| Intoxication | 2 | 1 | 1 | 0 | 0 | 0 |
|  | (0,4%) | (2,5%) | (0,5%) | (0,0%) | (0,0%) | (0,0%) |
| Neurological | 2 | 0 | 0 | 2 | 0 | 0 |
|  | (0,4%) | (0,0%) | (0,0%) | (2,6%) | (0,0%) | (0,0%) |
| Medical Device Complication | 5 | 1 | 2 | 1 | 0 | 1 |
|  | (1,1%) | (2,5%) | (1,0%) | (1,3%) | (0,0%) | (1,0%) |
| Stomatology | 1 | 0 | 0 | 0 | 0 | 1 |
|  | (0,2%) | (0,0%) | (0,0%) | (0,0%) | (0,0%) | (1,0%) |
| Urologic | 1 | 0 | 0 | 0 | 0 | 1 |
|  | (0,2%) | (0,0%) | (0,0%) | (0,0%) | (0,0%) | (1,0%) |
| Total | 463 | 40 | 198 | 78 | 43 | 104 |
|  | (100,0%) | (100,0%) | (100,0%) | (100,0%) | (100,0%) | (100,0%) |

Supplemental Table 7: 3MM in patients with advanced lobular vs. non-lobular cancer (Chi-Square Test: p = 0.18)

|  | **3-month-mortality** |
| --- | --- |
| Lobular cancer | 18/35 (51.4 %) |
| Non-lobular caner | 76/194 (39.2 %) |

Supplemental Table 8: Significant differences in 3MM according to subtype among 35 patients with advanced lobular cancer (Fisher-Freeman-Halton Exact Test: p = 0.012)

|  | **3-month mortality** |
| --- | --- |
| Luminal A-like | 2/9 (22.2 %) |
| Luminal B-like/HER2-negative | 15/22 (68.2 %) |
| Luminal B-like/HER2-positive | 0/3 (0 %) |
| HER2-positive (non-luminal) | 0/0 (0 %) |
| Triple negative | 0/1 (0 %) |
